# Association Between Socioeconomic Status and Mortality Risk in CKM Stage 0–3 Patients: Analysis of Inflammatory Mediation

**DOI:** 10.1155/crp/8849559

**Published:** 2026-04-12

**Authors:** Wenlong Ding, Fachao Shi, Lei Fang, Qin Cui, Zheng Wang, Caoyang Fang

**Affiliations:** ^1^ Department of Cardiology, Xuancheng Hospital Affiliated to Wannan Medical College, Xuancheng People’ Hospital, Xuancheng, Anhui, 242000, China; ^2^ Department of Cardiology, Maanshan People’s Hospital, Maanshan Hospital Affiliated to Wannan Medical College, Maanshan, Anhui, 243000, China; ^3^ Department of Geriatrics Center, Tongling People’s Hospital, Tongling, Anhui, 244000, China, eurofinsa.com; ^4^ Department of Cardiology, The Second People’s Hospital of Hefei, Hefei Hospital Affiliated to Anhui Medical University, Hefei, Anhui, 230000, China, ahmu.edu.cn; ^5^ Department of Emergency, The First Affiliated Hospital of USTC, Division of Life Sciences and Medicine, University of Science and Technology of China, Hefei, Anhui, 230000, China, ustc.edu.cn

**Keywords:** all-cause mortality, cardiovascular–renal–metabolic syndrome, cardiovascular mortality, mediation analysis, socioeconomic status, systemic inflammation response index

## Abstract

**Objective:**

This study aimed to investigate whether socioeconomic status (SES) influences the risk of all‐cause and cardiovascular mortality among patients with cardiovascular–kidney–metabolic (CKM) syndrome stages 0–3 through the systemic inflammation response index (SIRI). The investigation utilized a nationally representative sample from the U.S. National Health and Nutrition Examination Survey (NHANES) database and employed mediation analysis for systematic assessment.

**Methods:**

Data were derived from NHANES surveys conducted between 1999 and 2018, enrolling adults who met the classification criteria for CKM stages 0–3. SES was defined by the stratified family income‐to‐poverty ratio (PIR), with SIRI serving as the mediator. The primary outcomes were all‐cause and cardiovascular mortality. Methodological approaches included weighted multivariable Cox regression, subgroup analyses, sensitivity analyses, and bootstrap‐based mediation analysis.

**Results:**

The study included 15,623 participants who were followed for a mean duration of 115 months, during which 1788 all‐cause deaths and 405 cardiovascular deaths were recorded. After comprehensive adjustment for potential confounders, each unit increase in PIR was associated with a significantly reduced risk of all‐cause mortality (HR = 0.88, 95% CI: 0.84–0.92) and cardiovascular mortality (HR = 0.85, 95% CI: 0.77–0.95). Participants in the high SES group demonstrated substantially lower risks for both all‐cause mortality (HR = 0.57, 95% CI: 0.47–0.69) and cardiovascular mortality (HR = 0.50, 95% CI: 0.34–0.73) compared to their low SES counterparts. Notably, mediation analysis revealed that SIRI accounted for 63.67% of the association between SES and all‐cause mortality, and 60.45% of the association between SES and cardiovascular mortality after full adjustment for confounding variables.

**Conclusion:**

SES significantly impacts the risk of all‐cause and cardiovascular mortality among patients with CKM stages 0–3, with a substantial portion of this effect mediated through systemic inflammation as measured by SIRI. These findings suggest that comprehensive interventions targeting both socioeconomic conditions and chronic inflammation may effectively enhance long‐term health outcomes in this vulnerable population.

## 1. Introduction

The global burden of noncommunicable diseases is increasingly dominated by the interconnected pathologies affecting cardiovascular, renal, and metabolic systems. Contemporary epidemiological trends, driven by demographic transitions, environmental factors, and lifestyle modifications, have led to an escalating prevalence of multisystem disease presentations [1]. The emerging cardiovascular–kidney–metabolic (CKM) syndrome paradigm provides a unified framework for understanding the pathophysiological interconnections between CVD, chronic kidney disease, and metabolic dysfunction [2]. Numerous epidemiological studies have demonstrated that patients with CKM face significantly higher mortality risks compared to those with single‐category diseases—a disparity particularly pronounced among older adults and individuals with pre‐existing chronic conditions [3–5]. Consequently, early identification and precise prediction of multiple risk factors for adverse outcomes in CKM patients have emerged as priority research areas within the international scientific community.

Socioeconomic position functions as a fundamental upstream determinant of health trajectories and life expectancy. This multidimensional construct, characterized by income distribution, educational achievement, and professional standing, demonstrates consistent associations with chronic disease incidence and progression patterns [6, 7]. Within the CKM population specifically, low socioeconomic status (SES) frequently correlates with limited access to healthcare resources, insufficient health literacy, and suboptimal health behaviors, all factors that can precipitate and exacerbate cardiovascular and renal injuries [8, 9]. Previous large‐scale cohort investigations have consistently identified an inverse association between SES and cardiovascular mortality risk [10, 11]. Nevertheless, the biological mechanisms through which SES influences long‐term mortality outcomes remain incompletely elucidated.

Inflammation plays a central and integrative role in the pathogenesis and progression of various chronic diseases, including cardiovascular, renal, and metabolic disorders. Chronic inflammatory processes provide a common pathophysiological foundation for the development of cardiovascular–renal–metabolic comorbidities by promoting atherosclerosis, glomerulosclerosis, and insulin resistance [12–14]. Recently, the systemic inflammation response index (SIRI), a composite marker derived from peripheral blood leukocyte subtypes, has emerged as a reliable prognostic indicator across multiple disease states [15–17]. Notably, individuals with lower SES tend to exhibit more pronounced states of chronic inflammation. Socioeconomic disadvantage may trigger inflammatory activation and immune dysregulation through chronic psychological stress, adverse environmental exposures, and unhealthy lifestyle patterns, ultimately increasing the risk of adverse clinical outcomes [18, 19].

It is particularly noteworthy that a potential mediational pathway may exist linking SES, inflammation, and mortality risk. Several studies suggest that low SES, by facilitating the accumulation of physiological stress over time, activates inflammatory pathways that subsequently influence both cardiovascular and all‐cause mortality risk [20, 21]. However, verification of these mediational mechanisms in patients across varying stages of CKM syndrome remains inadequate, especially in nationally representative samples. The U.S. National Health and Nutrition Examination Survey (NHANES), with its longitudinal mortality data and comprehensive collection of biological and socioeconomic variables, offers an exceptional opportunity to investigate these relationships.

Therefore, utilizing the extensive data from the NHANES cohort, this study aims to systematically evaluate the association between SES and the risks of all‐cause and cardiovascular mortality among patients with CKM stages 0–3 and to further elucidate the potential mediating role of SIRI in these associations. The findings are expected to provide robust theoretical support and evidence‐based insights to inform disease prevention strategies and public health policy development.

## 2. Methods

### 2.1. Study Design and Population

Our study utilized data from the NHANES, a comprehensive population‐based surveillance program administered by the Centers for Disease Control and Prevention. This program implements complex probability sampling methodology to obtain representative data characterizing the civilian, noninstitutionalized U.S. population. Conducted biennially, NHANES comprehensively assesses the health and nutritional status of civilians across the country. The study protocol has received full approval from the Ethics Review Board of the National Center for Health Statistics, and all participants provided written informed consent before enrollment. Our investigation utilized de‐identified, publicly available NHANES data for secondary analysis, thereby presenting minimal privacy risk to participants and meeting criteria for exemption from additional ethical review.

**FIGURE 1:**
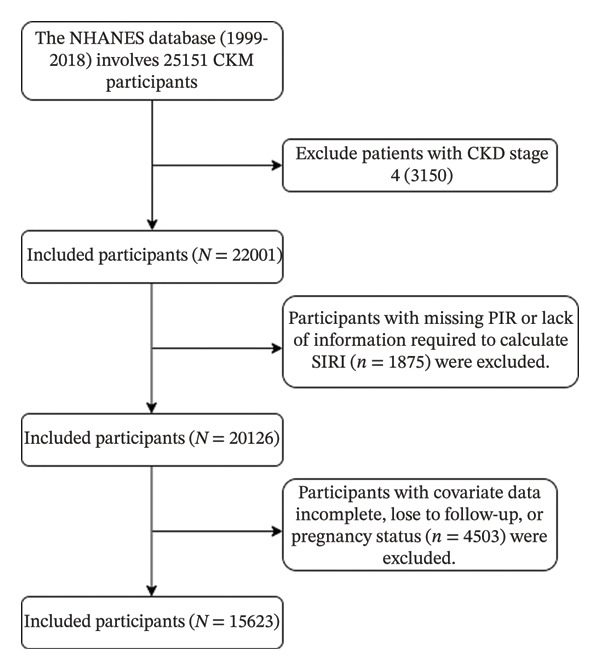
Study flowchart.

For this study, we initially considered 25,151 participants from the NHANES cycles conducted between 1999 and 2018. We applied the following inclusion criteria [1]: age ≥ 20 years [2]; complete data on poverty income ratio (PIR), relevant laboratory measurements, and mortality follow‐up; and [3] classification within CKM stages 0–3. We excluded participants based on the following criteria [1]: classification as CKM stage 4 (*n* = 3150) [2]; missing PIR data or insufficient information to calculate the SIRI (*n* = 1875); and [3] incomplete covariate data, loss to follow‐up, or pregnancy (*n* = 4503). After applying these criteria, 15,623 participants remained eligible for inclusion in the final analysis (Figure 1).

### 2.2. Assessment of Household PIR and SES

The PIR is calculated according to guidelines established by the U.S. Department of Health and Human Services and serves as a standardized measure for assessing participants’ SES [22]. This ratio is specifically determined by dividing a household’s total annual income by the federal poverty threshold corresponding to the relevant year. In instances where income is reported as a range rather than a precise figure, the midpoint of the reported range is utilized for calculation purposes. Notably, due to data confidentiality and protection policies, all PIR values equal to or exceeding 5.0 are uniformly recorded as 5.0 in the dataset. For analytical purposes in this study, household income levels were stratified into three distinct categories: low, middle, and high, using PIR cut‐off points of 1.3 and 3.5, respectively, in accordance with established methodological precedents [23].

### 2.3. Definition of Cardiovascular–Renal–Metabolic Syndrome

The CKM staging classification was defined in accordance with the latest consensus guidelines, utilizing disease history, laboratory parameters, and physical examination indicators collected from the NHANES database [24, 25]. The staging criteria were operationalized as follows: CKM Stage 0: healthy individuals presenting with no underlying diseases or identifiable risk factors. CKM Stages 1–3: characterized by the presence of metabolic syndrome, early kidney injury, or initial cardiovascular dysfunction. Specific diagnostic indicators include hypertension, diabetes mellitus, obesity, estimated glomerular filtration rate (eGFR), urinary albumin excretion, and other relevant biomarkers. CKM Stage 4: established clinical CVD occurring within the context of CKM syndrome, encompassing coronary heart disease, heart failure, cerebrovascular accident, peripheral arterial disease, and atrial fibrillation. Comprehensive and detailed criteria for each stage are provided in Supporting Table S1.

### 2.4. Definition of SIRI

SIRI represents a composite inflammatory biomarker derived from peripheral blood cellular components. This index quantifies systemic inflammatory burden through the mathematical relationship: SIRI = (neutrophil count × monocyte count)/lymphocyte count, with all measurements expressed as 10^9 cells per liter. The index is derived by multiplying the absolute neutrophil count by the absolute monocyte count, then dividing this product by the absolute lymphocyte count (SIRI = neutrophil count × monocyte count/lymphocyte count). All cellular measurements are standardized in units of 10^9/L. The values for each leukocyte subpopulation were extracted from comprehensive NHANES clinical laboratory data. This composite inflammatory index serves as a validated biomarker for quantifying an individual’s degree of systemic chronic inflammation [26].

### 2.5. Death‐Related Information

The primary outcomes of this investigation were all‐cause mortality and CVD mortality. Mortality status was ascertained through probabilistic matching between NHANES participants from 1999 to 2018 and death records maintained in the National Death Index. CVD mortality specifically was identified using International Classification of Diseases, 10th Revision (ICD‐10) diagnostic codes, encompassing I00–I09, I11, I13, I20–I51, and I60–I69. Due to the complex stratified sampling design of NHANES and the sequential nature of participant enrollment across multiple survey cycles, it was not methodologically feasible to calculate an accurate median follow‐up duration for the entire cohort. Consequently, the follow‐up period in this study was operationally defined as the interval from initial enrollment until either the date of death or the administrative end date of December 31, 2019, whichever occurred first.

### 2.6. Covariates

This study incorporated a comprehensive range of demographic and clinical covariates. Demographic variables included age, sex, and race/ethnicity (categorized as non‐Hispanic White, non‐Hispanic Black, Mexican American, and other), as well as education level and marital status. Lifestyle factors encompassed smoking status and alcohol consumption patterns. Smoking status was stratified into three distinct categories [1]: never smokers (individuals who had smoked fewer than 100 cigarettes in their lifetime) [2]; former smokers (those who had smoked more than 100 cigarettes but subsequently discontinued); and [3] current smokers (individuals who had smoked more than 100 cigarettes and continued to smoke). Alcohol consumption was classified into five categories: never drinkers (consumed alcohol fewer than 12 times in their lifetime); former drinkers (did not consume alcohol in the past year but previously consumed ≥ 12 drinks, or consumed alcohol ≥ 12 times in the past year but were not currently drinking); heavy drinkers (consumed ≥ 3 drinks per day for women or ≥ 4 for men, or engaged in binge drinking on ≥ 5 days per month); moderate drinkers (consumed ≤ 2 drinks per day for women or ≤ 3 for men, or engaged in binge drinking on ≥ 2 days per month); and mild drinkers (individuals not classified within the aforementioned categories). Anthropometric and clinical parameters included body mass index (BMI), hypertension, diabetes, and medication usage (specifically antihypertensive, antidiabetic, and lipid‐lowering agents). BMI was calculated as weight in kilograms divided by height in meters squared (kg/m^2^) and categorized as normal (< 25 kg/m^2^), overweight (25–30 kg/m^2^), or obese (> 30 kg/m^2^). Additionally, an extensive panel of laboratory assessments was conducted, comprising measurements of renal function (creatinine, uric acid, blood urea nitrogen, and eGFR), hematological parameters (lymphocyte, neutrophil, and monocyte counts), glycemic indices (HbA1c and fasting glucose), and lipid profile components (triglycerides, total cholesterol, high‐density lipoprotein, and low‐density lipoprotein).

### 2.7. Statistical Analysis

All data analyses in this study were performed using *R* software (Version 4.3.2, https://www.r-project.org). SES was categorized into low, middle, and high groups based on PIR. Continuous variables are presented as mean (SE), while categorical variables are shown as frequency (%).

This study strictly followed NHANES analysis guidelines for handling complex sampling design and survey weights [27]. Since our analysis combined data from multiple NHANES survey cycles (1999–2018), we appropriately adjusted weights according to NHANES guidelines. Specifically, for full sample analyses, we used the WTMEC2YR (Medical Examination Center examination weight) variable, dividing it by the number of survey cycles used (10 cycles) to create a new weight variable. For mortality analyses, we used the public‐use linked mortality files provided by NHANES and applied the corresponding mortality weights (WTPAF2YR) with appropriate adjustments. In all statistical analyses, we incorporated primary sampling units (PSU), stratification variables (SDMVSTRA), and adjusted weights to correctly estimate standard errors and confidence intervals (CIs). Specifically: (1) In descriptive statistics, weighted percentages and means were calculated by applying the adjusted survey weights. (2) In Cox proportional hazards regression models, we used the “survey” package and “svycoxph” function in *R* software to incorporate complex sampling design and corresponding weights. (3) For mediation analysis, we adopted a weight‐based approach, passing the adjusted survey weights through the weight parameter in the “mediation” package. (4) In subgroup analyses and sensitivity analyses, we maintained the same weight application strategy to ensure consistency and comparability of results. (5). All *p* values were based on survey design‐adjusted Wald tests, and all CIs were calculated by considering sampling weights and design effects. This comprehensive weight application strategy ensures that our study results can be appropriately generalized to the U.S. general population while accurately estimating the confidence in the associations between SES, SIRI, and mortality in CKM stage 0–3 patients.

To investigate the association between SES and all‐cause and cardiovascular mortality risk among participants with CKM stages 0–3, we applied multivariable weighted Cox proportional hazards models. To control for confounding factors, models with different levels of adjustment were constructed: Model 1 was unadjusted; Model 2 adjusted for age, sex, and race/ethnicity; Model 3 further adjusted for marital status, education level, smoking status, alcohol consumption, and BMI; and Model 4 additionally included eGFR, HbA1c, triglycerides, HDL, LDL, as well as medication use for hypertension, dyslipidemia, and diabetes.

Restricted cubic spline (RCS) analysis was employed to evaluate potential linear or nonlinear relationships between PIR and the risk of all‐cause and cardiovascular mortality among participants with CKM syndrome stages 0–3. To elucidate underlying mechanisms, we conducted a comprehensive mediation analysis to quantify the indirect effect of the SIRI on the association between SES and mortality outcomes, reporting both the magnitude and proportion of the mediating effect. Furthermore, we performed stratified analyses across multiple predefined subgroups, including age (< 65 vs. ≥ 65 years), BMI categories (< 25, 25–30, and > 30 kg/m^2^), sex, presence or absence of hypertension or diabetes, and race/ethnicity (Mexican American, non‐Hispanic White, non‐Hispanic Black, and other racial/ethnic groups). To assess model robustness, we conducted several sensitivity analyses: re‐examining the association between SES and both mortality outcomes after excluding deaths that occurred within the first 2 years of follow‐up; excluding participants with self‐reported history of cancer to minimize potential confounding effects; and implementing unweighted multivariable logistic regression as an alternative analytical approach to evaluate the relationship between SES and mortality risk. Additionally, Kaplan–Meier survival curves were generated, and log‐rank tests were performed to compare all‐cause and cardiovascular mortality risks across different SES groups. Throughout all analyses, a two‐sided *p* value < 0.05 was considered statistically significant.

## 3. Results

### 3.1. General Characteristics of Participants

This study included a total of 15,624 participants with CKM syndrome stages 0–3. The cohort had a mean age of 45.62 (± 0.24) years, with males constituting 48.78% of the sample. Regarding socioeconomic distribution, the low SES group accounted for 45.63% of participants, while the high SES group comprised 50.88%. Among participants with CKM stages 0–3, those in the high SES category were predominantly characterized by older age, non‐Hispanic White ethnicity, and male gender. This group also demonstrated significantly higher educational attainment and lower divorce rates compared to their counterparts in other SES categories. Notably, both all‐cause and cardiovascular mortality rates were significantly lower among participants in the high SES group. Comprehensive baseline characteristics of the study population are presented in Table 1.

**TABLE 1:** Baseline characteristics of the study participants according to SES.

Variables	Total	Low	Middle	High	*p* value
Age, mean (SE)	45.62 (0.24)	41.74 (0.37)	45.35 (0.33)	47.51 (0.32)	< 0.0001
Creatinine, mean (SE)	76.14 (0.28)	73.62 (0.48)	76.14 (0.49)	77.23 (0.33)	< 0.0001
UA, mean (SE)	322.84 (0.91)	316.60 (1.45)	323.90 (1.46)	324.68 (1.31)	< 0.0001
BUN, mean (SE)	4.69 (0.02)	4.35 (0.04)	4.66 (0.03)	4.87 (0.03)	< 0.0001
TG, mean (SE)	1.35 (0.01)	1.38 (0.02)	1.35 (0.01)	1.33 (0.01)	0.04
TC, mean (SE)	5.05 (0.01)	4.94 (0.02)	5.03 (0.02)	5.12 (0.02)	< 0.0001
HDL, mean (SE)	1.40 (0.01)	1.33 (0.01)	1.39 (0.01)	1.45 (0.01)	< 0.0001
LDL, mean (SE)	3.03 (0.01)	2.98 (0.02)	3.02 (0.02)	3.06 (0.02)	0.01
PIR, mean (SE)					
BMI, mean (SE)	28.61 (0.08)	28.94 (0.15)	28.88 (0.12)	28.26 (0.13)	< 0.001
eGFR, mean (SE)	96.26 (0.31)	102.12 (0.50)	97.07 (0.41)	93.11 (0.38)	< 0.0001
Lymphocyte, mean (SE)	2.00 (0.01)	2.14 (0.02)	2.02 (0.02)	1.92 (0.01)	< 0.0001
Monocyte, mean (SE)	0.53 (0.00)	0.55 (0.00)	0.53 (0.00)	0.52 (0.00)	< 0.0001
Neutrophils, mean (SE)	3.95 (0.02)	4.19 (0.04)	4.03 (0.03)	3.78 (0.03)	< 0.0001
FPG, mean (SE)	5.73 (0.02)	5.82 (0.03)	5.77 (0.03)	5.66 (0.02)	< 0.001
HbA1c, mean (SE)	5.51 (0.01)	5.59 (0.02)	5.55 (0.02)	5.45 (0.01)	< 0.0001
Sex, % (SE)					< 0.0001
Female	51.22 (0.01)	54.37 (0.86)	52.16 (0.65)	49.12 (0.63)	
Male	48.78 (0.01)	45.63 (0.86)	47.84 (0.65)	50.88 (0.63)	
Race, % (SE)					< 0.0001
Mexican American	8.09 (0.00)	16.64 (1.31)	9.70 (0.75)	3.13 (0.28)	
Non‐Hispanic Black	9.79 (0.00)	15.85 (1.12)	11.58 (0.73)	5.76 (0.45)	
Non‐Hispanic White	70.76 (0.02)	50.98 (2.21)	66.77 (1.38)	82.42 (0.84)	
Others	11.37 (0.01)	16.53 (1.20)	11.95 (0.78)	8.69 (0.58)	
Marital, % (SE)					< 0.0001
Divorced	10.12 (0.00)	12.48 (0.80)	11.11 (0.53)	8.33 (0.54)	
Married	57.31 (0.02)	35.44 (1.12)	52.80 (1.00)	70.29 (0.97)	
Never married	17.91 (0.01)	26.76 (1.15)	19.14 (0.82)	13.14 (0.65)	
Others	14.65 (0.01)	25.32 (0.93)	16.95 (0.66)	8.24 (0.56)	
Education, % (SE)					< 0.0001
High school or equivalent	23.68 (0.01)	28.17 (1.04)	30.26 (0.89)	16.53 (0.71)	
Less than high school	15.28 (0.01)	35.25 (1.08)	17.34 (0.68)	5.07 (0.42)	
Some college or above	61.04 (0.02)	36.58 (1.18)	52.41 (1.02)	78.40 (0.88)	
Smoke, % (SE)					< 0.0001
Never	54.66 (0.01)	48.67 (1.43)	52.66 (1.04)	58.82 (1.04)	
Former	24.41 (0.01)	17.39 (0.79)	24.64 (0.75)	27.24 (0.95)	
Now	20.93 (0.01)	33.94 (1.32)	22.70 (0.81)	13.94 (0.70)	
Alcohol, % (SE)					< 0.0001
Never	10.57 (0.01)	16.77 (1.01)	11.86 (0.63)	6.88 (0.52)	
Former	12.91 (0.01)	16.73 (0.74)	14.28 (0.65)	10.18 (0.61)	
Mild	36.98 (0.01)	23.59 (0.93)	33.20 (0.74)	45.74 (0.98)	
Moderate	17.91 (0.01)	13.22 (0.65)	16.88 (0.67)	20.73 (0.66)	
Heavy	21.63 (0.01)	29.69 (1.06)	23.78 (0.82)	16.46 (0.72)	
Hypertension, % (SE)					0.28
No	66.14 (0.02)	67.54 (0.94)	65.39 (0.79)	66.12 (0.90)	
Yes	33.86 (0.01)	32.46 (0.94)	34.61 (0.79)	33.88 (0.90)	
Diabetes, % (SE)					< 0.0001
No	74.07 (0.02)	72.00 (0.86)	73.38 (0.78)	75.49 (0.84)	
Yes	11.71 (0.00)	13.83 (0.58)	13.10 (0.57)	9.70 (0.53)	
Borderline	14.22 (0.01)	14.17 (0.70)	13.52 (0.63)	14.81 (0.66)	
All‐cause mortality, % (SE)					< 0.0001
No	91.85 (0.02)	89.28 (0.59)	90.26 (0.53)	94.22 (0.35)	
Yes	8.15 (0.00)	10.72 (0.59)	9.74 (0.53)	5.78 (0.35)	
Cardiovascular mortality, % (SE)					< 0.0001
No	98.24 (0.02)	97.56 (0.28)	97.83 (0.23)	98.86 (0.15)	
Yes	1.76 (0.00)	2.44 (0.28)	2.17 (0.23)	1.14 (0.15)	
Antidiabetic drugs, % (SE)					< 0.001
No	93.87 (0.02)	93.56 (0.38)	92.82 (0.45)	94.85 (0.40)	
Yes	6.13 (0.00)	6.44 (0.38)	7.18 (0.45)	5.15 (0.40)	
Antihypertensive drugs, % (SE)					< 0.0001
No	77.83 (0.02)	81.68 (0.78)	76.79 (0.83)	77.00 (0.79)	
Yes	22.17 (0.01)	18.32 (0.78)	23.21 (0.83)	23.00 (0.79)	
Antihyperlipidemic drugs, % (SE)					< 0.0001
No	87.28 (0.02)	90.66 (0.59)	88.17 (0.62)	85.12 (0.59)	
Yes	12.72 (0.00)	9.34 (0.59)	11.83 (0.62)	14.88 (0.59)	

*Note:* Data are presented as mean (SE) or *n* (%); HbA1c: glycosylated hemoglobin.

Abbreviations: BMI, body mass index; BUN, blood urea nitrogen; eGFR, estimated glomerular filtration rate; FPG, fasting plasma glucose; HDL, high‐density lipoprotein; LDL, low‐density lipoprotein; PIR, poverty income ratio; SES, socioeconomic status; TG, triglyceride; TC, total cholesterol; UA, uric acid.

### 3.2. SES and Risk of All‐Cause and Cardiovascular Mortality

Over a mean follow‐up period of 115 months, we documented 1788 all‐cause deaths and 405 cardiovascular‐specific deaths. Multivariable Cox proportional hazards regression analysis revealed that, compared with the high SES group, the low SES group exhibited substantially elevated risks of both all‐cause mortality (adjusted hazard ratio [HR] = 0.57, 95% CI: 0.47–0.69, *p* < 0.0001) and cardiovascular mortality (adjusted HR = 0.50, 95% CI: 0.34–0.73, *p* < 0.0001). Notably, the inverse association between SES and mortality risk persisted after comprehensive adjustment for multiple potential confounding variables, underscoring the robust independent nature of this relationship (Table 2). RCS analysis (Figure 2(a)) demonstrated a significant nonlinear relationship between PIR and all‐cause mortality risk among participants with CKM syndrome stages 0–3 (nonlinearity *p* < 0.05). In contrast, the relationship between PIR and cardiovascular mortality risk exhibited a linear pattern (Figure 2(b), nonlinearity *p* > 0.05). Multivariable‐adjusted Kaplan–Meier survival curves with corresponding log‐rank tests (Figure 3) further corroborated these findings, clearly illustrating that both all‐cause and cardiovascular mortality risks were significantly higher in the low SES group compared to the high SES group (*p* < 0.05).

**TABLE 2:** Association of the SES and all‐cause and cardiovascular mortality in the population with CKM syndrome stages 0–3.

Variables	Model 1		Model 2		Model 3		Model 4	
	HR (95%CI)	*p*	HR (95%CI)	*p*	HR (95%CI)	*p*	HR (95%CI)	*p*
*All-cause mortality*								
PIR	0.84 (0.81.0.87)	< 0.0001	0.80 (0.77.0.83)	< 0.0001	0.88 (0.84.0.92)	< 0.0001	0.88 (0.84.0.92)	< 0.0001

*SES*								
Low	Ref	Ref	Ref	Ref	Ref	Ref	Ref	Ref
Middle	0.87 (0.74.1.02)	0.08	0.62 (0.54.0.72)	< 0.0001	0.73 (0.64.0.83)	< 0.0001	0.73 (0.63.0.83)	< 0.0001
High	0.50 (0.42.0.59)	< 0.0001	0.40 (0.34.0.47)	< 0.0001	0.57 (0.47.0.69)	< 0.0001	0.57 (0.47.0.69)	< 0.0001
P for trend	0.984		< 0.001		0.007		0.005	

*Cardiovascular mortality*								
PIR	0.81 (0.75.0.87)	< 0.0001	0.76 (0.69.0.83)	< 0.0001	0.86 (0.77.0.95)	0.004	0.85 (0.77.0.95)	0.004

*SES*								
Low	Ref	Ref	Ref	Ref	Ref	Ref	Ref	Ref
Middle	0.85 (0.62.1.16)	0.31	0.60 (0.44.0.81)	< 0.001	0.73 (0.54.1.00)	0.05	0.70 (0.52.0.95)	0.02
High	0.42 (0.31.0.58)	< 0.0001	0.33 (0.24.0.47)	< 0.0001	0.51 (0.35.0.76)	< 0.001	0.50 (0.34.0.73)	< 0.001
P for trend	0.951	0.147	0.352	0.234	0.951	0.147	0.352	0.234

*Note:* Model 1: No adjustments made; Model 2: Adjusted for age, sex, and race; Model 3: Adjusted for age, sex, race, BMI, marital, education, smoke, and alcohol; Model 4: Adjusted for age and sex, race, BMI, marital, education, smoke, alcohol, eGFR, HbA1c, TG, HDL, LDL, drugs for hypertension, hyperlipidemia, and diabetes.

Abbreviations: CI, confidence interval; HR, hazard ratio; Ref, reference.

FIGURE 2The RCS analysis of SES and all‐cause and cardiovascular mortality in the population with CKM syndrome stages 0–3. CKM: cardiovascular–kidney–metabolic; RCS: restricted cubic spline; SES: socioeconomic status; (a) all‐cause mortality; (b) cardiovascular mortality.

**Figure:**
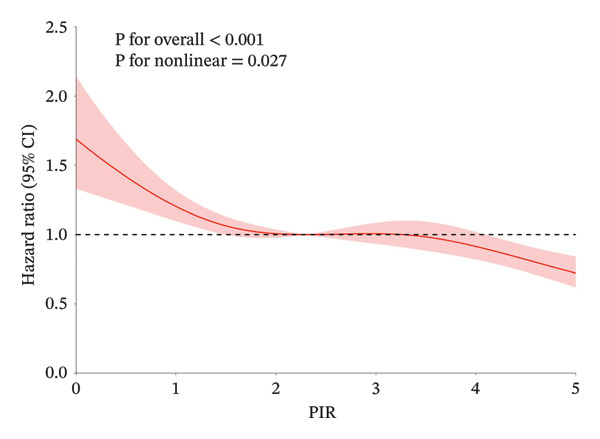
(a)

**Figure:**
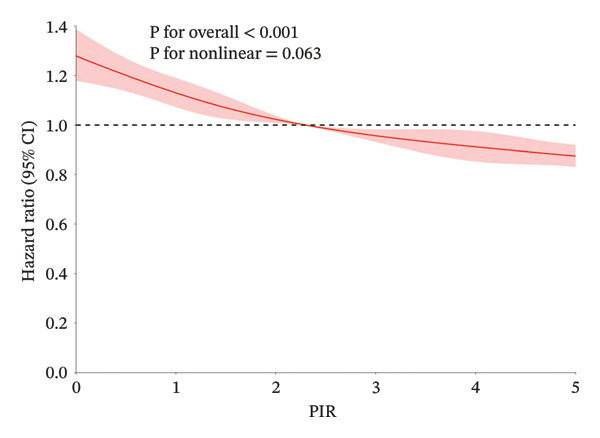
(b)

FIGURE 3Kaplan–Meier survival curves for patients with CKM syndrome stages 0–3 in different SES groups. *p* < 0.0001 by the log‐rank test. CKM: cardiovascular–kidney–metabolic; SES: socioeconomic status; (a) all‐cause mortality; (b) cardiovascular mortality.

**Figure:**
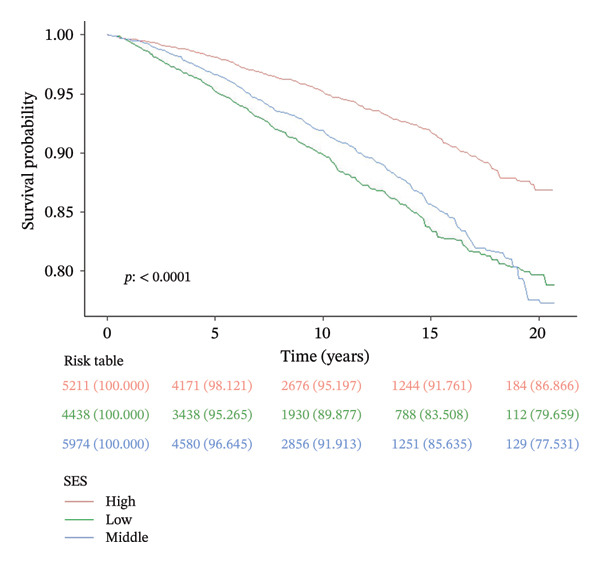
(a)

**Figure:**
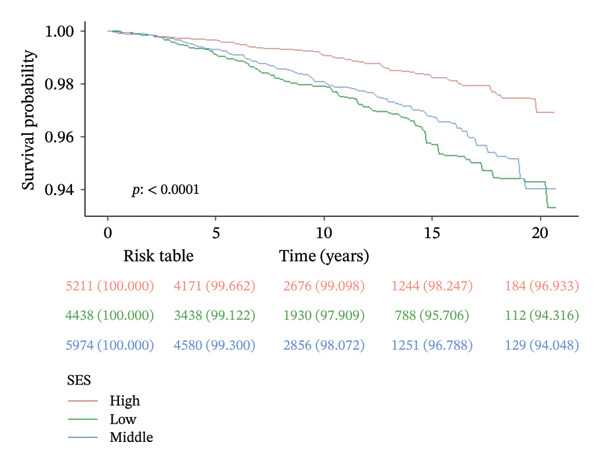
(b)

### 3.3. Subgroup Analysis and Sensitivity Analysis

We performed comprehensive subgroup analyses stratified by key demographic and clinical variables, including age, sex, race/ethnicity, BMI, hypertension status, and diabetes status. These analyses revealed a statistically significant interaction between PIR and age in relation to all‐cause mortality risk (*p* < 0.05), indicating that the association between SES and mortality varied across age groups. In contrast, no significant interactions were observed between baseline PIR and any stratification variables regarding cardiovascular mortality risk (all interaction *p* > 0.05) (Figure 4).

FIGURE 4Forest plot for subgroup analyses. (a) All‐cause mortality; (b) cardiovascular mortality.

**Figure:**
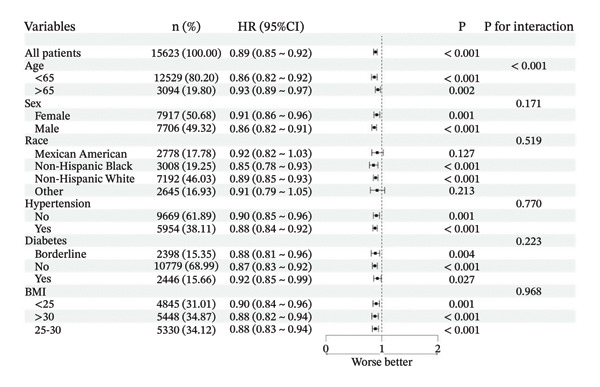
(a)

**Figure:**
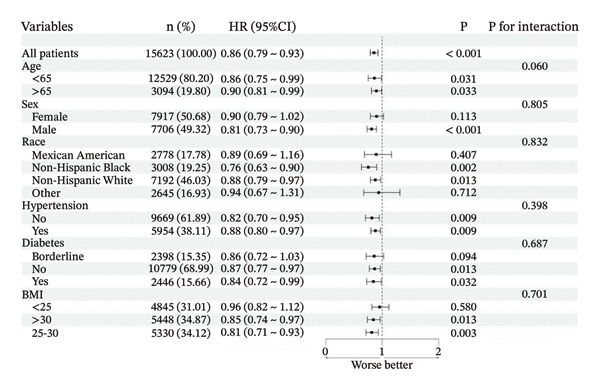
(b)

To evaluate model robustness, we conducted several sensitivity analyses. First, we excluded participants with CKM syndrome stages 0–3 who died within the initial 2 years of follow‐up; notably, the associations between SES and both all‐cause and cardiovascular mortality risks remained consistent with our primary findings (Table S2). Second, after excluding participants who self‐reported a cancer diagnosis at baseline and further adjusting for potential confounders, the relationship between SES and mortality outcomes did not demonstrate substantial alterations (Table S3). Furthermore, when comparing weighted and unweighted analytical approaches, we observed that the weighted HRs were modestly lower; yet, all associations maintained both statistical significance and directional consistency (Table S4). Given the complex multistage probability sampling design employed in the NHANES, weighted analysis methodology provides more accurate population‐representative estimates.

### 3.4. Mediation Analysis

**FIGURE 5:**
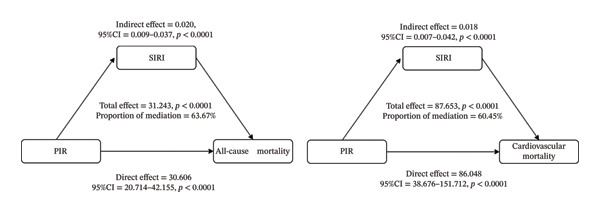
Mediating effect analysis of SIRI between SES and risk of all‐cause and cardiovascular mortality in CKM patients. CKM: cardiovascular–kidney–metabolic; SES: socioeconomic status; (a) all‐cause mortality; (b) cardiovascular mortality.

Formal mediation analysis demonstrated that the SIRI served as a significant mediator in the relationship between SES and mortality outcomes. Specifically, regarding the association between PIR and all‐cause mortality risk among participants with CKM syndrome stages 0–3, the indirect effect mediated through SIRI constituted 63.67% of the total effect (95% CI:0.288–1.072, *p* < 0.0001). Similarly, for cardiovascular mortality risk, SIRI mediated 60.45% of the total effect (95% CI: 0.231–0.973, *p* < 0.0001). These findings indicate that systemic inflammation, as quantified by SIRI, represents a substantial biological pathway through which socioeconomic disparities influence mortality outcomes. Comprehensive mediation analysis results are illustrated in Figure 5.

## 4. Discussion

Drawing upon a nationally representative sample from the NHANES, this study systematically investigated the association between SES and the risk of all‐cause and cardiovascular mortality among individuals with CKM syndrome stages 0–3, while examining the mediating role of the SIRI. The analysis included 15,624 participants with CKM stages 0–3, with a mean age of 45.62 years, of whom 48.78% were male. SES demonstrated relatively even distribution across the overall sample; notably, participants in the high SES group were predominantly older, non‐Hispanic White males, characterized by higher educational attainment and lower divorce rates.

During the follow‐up period, we documented 1788 all‐cause deaths and 405 cardiovascular‐specific deaths. Multivariable Cox regression analysis revealed that lower SES was significantly associated with increased risks of both all‐cause and cardiovascular mortality. This association remained robust after comprehensive adjustment for multiple covariates (adjusted HRs were 0.57 and 0.50, respectively; both *p* < 0.0001). The consistency of these findings was further substantiated through extensive subgroup and sensitivity analyses. Importantly, mediation analysis demonstrated that SIRI accounted for 63.67% and 60.45% of the total effect of SES on all‐cause and cardiovascular mortality risk, respectively, highlighting the substantial mediating role of inflammation in the pathway linking socioeconomic disparities to mortality outcomes.

This investigation contributes to the existing literature by confirming the relationship between SES and mortality risk among individuals with CKM stages 0–3. Contemporary epidemiological evidence consistently identifies socioeconomic position as a primary social determinant of mortality outcomes [28]. Over recent decades, numerous cohort studies conducted across Europe, Asia, and the Americas have consistently demonstrated that populations with lower SES experience significantly worse health outcomes, particularly regarding cardiovascular mortality, compared to their higher SES counterparts [29, 30].

SES influences mortality risk through multiple pathways, including health behaviors (such as smoking patterns, dietary habits, and physical activity levels), prevalence of underlying diseases, and capacity to manage acute medical events. Several investigations have reported that the adverse effects of socioeconomic disadvantage are more pronounced during the early stages of cardiovascular–renal–metabolic comorbidity [31, 32]. Our findings further suggest that the predictive value of SES for mortality risk among individuals with CKM stages 0–3 remains independent of demographic, behavioral, metabolic, and chronic comorbidity factors. This not only corroborates previous conclusions but also provides a practical social attribute indicator for risk stratification in high‐risk populations.

Regarding the association between SIRI and mortality risk among individuals with CKM stages 0–3, existing evidence indicates that chronic inflammation represents a common pathway linking multiple chronic noncommunicable diseases to elevated mortality risk. Peripheral blood inflammatory cell counts—including neutrophils, lymphocytes, and monocytes—are widely considered sensitive indicators of systemic inflammatory burden [33]. SIRI integrates these cell types by quantifying their relative proportions, thereby providing a more comprehensive measure than single inflammatory markers for predicting mortality risk in patients with multiple comorbidities [34].

In recent years, large cohort and retrospective studies have demonstrated that SIRI consistently outperforms traditional markers in predicting all‐cause and cardiovascular mortality risk among populations with coronary heart disease, chronic kidney disease, and diabetes. Furthermore, combining SIRI with established inflammatory markers (such as C‐reactive protein) can enhance the precision of risk stratification [35, 36]. Our investigation further established that, even after rigorous stratification and adjustment for confounding variables, elevated SIRI remained strongly associated with both all‐cause and cardiovascular mortality in early‐stage CKM patients. This finding underscores the persistent adverse impact of the inflammatory phenotype on the longitudinal progression of chronic diseases. Notably, SIRI is easily implementable, cost‐effective, and holds significant potential for widespread clinical application.

Regarding the mediating role of SIRI in the relationship between SES and mortality risk among CKM stage 0–3 patients, this study represents the first systematic quantification of this mechanism in a nationally representative sample. Numerous investigations in social medicine, as well as the “biosocial pathways” theoretical framework, have proposed that the association between social disadvantage and adverse health outcomes is frequently mediated by biological factors, among which chronic inflammation represents one of the most robust mechanisms [37–39].

Populations experiencing socioeconomic disadvantage face cumulative exposure to chronic stressors, including financial insecurity, educational barriers, and adverse environmental conditions. Such sustained stress exposure triggers dysregulated inflammatory cascades and immune system activation through neuroendocrine pathways. These responses subsequently promote inflammatory mediator release and immune activation. Such inflammatory processes can induce endothelial dysfunction and accelerate atherosclerosis, while simultaneously contributing to injury of metabolic target organs including the glomeruli and pancreas [40]. In our analysis, SIRI accounted for more than 60% of the total effect of SES on both all‐cause and cardiovascular mortality risk, a proportion substantially higher than previously reported for single inflammatory mediators. This pronounced contribution may be closely related to the “inflammation‐driven” pathophysiological mechanisms characteristic of CKM populations, suggesting that the impact of biosocial pathways on mortality outcomes extends beyond independent or simple linear relationships.

Building upon the identified mediating mechanism of SIRI, it is worthwhile to further analyze why SIRI serves such a crucial bridging function in the association between SES and mortality risk. First, multiple social risk factors commonly observed in low SES populations, such as shorter educational duration, occupational exposures, and inadequate healthcare coverage, are directly linked to inflammatory activation, as confirmed by gene–environment interaction studies [41]. Second, inflammation functions both as a promoter of multimorbidity and an accelerator of disease progression. Chronic inflammation, through alterations in metabolic homeostasis, amplification of oxidative stress, and promotion of fibrotic processes, drives the transition from asymptomatic CKM status to end‐organ damage [42].

As a quantitative indicator of systemic inflammatory burden, elevated SIRI reflects the combined impact of social and biological stressors [43]. Moreover, SIRI integrates both immunosuppressive and activated immune states, providing a more comprehensive representation of immune dysregulation under the multifaceted influences of chronic disease. Therefore, incorporating SIRI into risk stratification algorithms for high‐risk SES groups (such as in community screening programs and health examinations) within national health management frameworks and clinical practice could facilitate early identification and proactive interventions, thereby improving chronic disease outcomes.

This study has several notable limitations. First, as NHANES employs an observational design, it cannot definitively establish causal relationships among SES, SIRI, and mortality risk, and the possibility of residual confounding persists. Second, SES assessment relied primarily on the PIR, which does not fully capture the multidimensional nature of SES, including educational attainment and occupational status. This relatively unidimensional evaluation may underestimate or overestimate the comprehensive impact of socioeconomic factors. Third, although SIRI incorporates routine blood cell parameters as an inflammatory marker, blood cell counts can be influenced by acute illnesses and medications, resulting in short‐term fluctuations that may not accurately reflect chronic inflammatory burden. Fourth, as the study included only patients with CKM stages 0–3, caution must be exercised when extrapolating these findings to advanced disease stages or other population groups. Fifth, certain covariates, such as smoking behavior and alcohol consumption, were self‐reported and may therefore be subject to information bias. Additionally, despite the NHANES survey’s implementation of a sophisticated sampling design with weighting adjustments, the sample size for specific subgroups remained relatively limited, potentially affecting the statistical power of certain subgroup analyses. Finally, while the mediation analysis revealed a statistically significant pathway via SIRI, the underlying biological mechanisms require further validation through basic science and interventional research approaches.

In conclusion, this study systematically elucidates the important pathway through which SES influences all‐cause and cardiovascular mortality risk via the SIRI in a nationally representative cohort of individuals with CKM stages 0–3. These findings enrich our understanding of risk factors in populations with CKM multimorbidity. The results suggest that future health management strategies for chronic disease populations should emphasize dual interventions targeting both socioeconomic determinants and chronic inflammation, thereby providing a theoretical and empirical foundation for more precise and effective public health and clinical prevention approaches.

**Nomenclature**
CKM: Cardiovascular–kidney–metabolic syndrome
NHANES: National Health and Nutrition Examination Survey
PIR: Poverty income ratio
BMI: Body mass index
FPG: Fasting plasma glucose
HbA1c: Glycosylated hemoglobin
UA: Uric acid
BUN: Blood urea nitrogen
TG: Triglyceride
TC: Total cholesterol
HDL: High‐density lipoprotein
LDL: Low‐density lipoprotein
eGFR: Estimated glomerular filtration rate
SES: Socioeconomic status
SIRI: Systemic inflammation response index
CVD: Cardiovascular disease
RCS: Restricted cubic spline

## Author Contributions

Wenlong Ding was responsible for study design and conceptualization, development of main data analysis methods, interpretation of results, and writing of the main manuscript; Caoyang Fang participated in the collection and validation of key data, execution of statistical analysis, and major manuscript revision; Fachao Shi undertook the processing and analysis of raw data, creation of all figures, and careful proofreading of manuscripts; Lei Fang participated in study concept formation, methodological design, data result validation, and manuscript review; Qin Cui supervised the entire project, coordinated research resources, verified the final results, and performed the final review of the manuscript; Zheng Wang participated in the overall study planning, provided important technical guidance, was responsible for study quality control, and contributed to manuscript refinement.

## Funding

The authors have nothing to report.

## Ethics Statement

The NHANES protocol has been approved by the NCHS Institutional Review Board, and this study does not contain any personally identifiable information. Therefore, the Ethics Committee of Maanshan People’s Hospital has exempted this study from ethical approval requirements.

## Conflicts of Interest

The authors declare no conflicts of interest.

## Supporting Information

Supporting Table S1. Definition of CKM.

Supporting Table S2. Sensitivity analysis excluding patients who died within 2 years prior to follow‐up.

Supporting Table S3. Sensitivity analyses excluded participants with self‐reported cancer diagnosis at baseline.

Supporting Table S4. Sensitivity analysis unweighted data analysis.

## Supporting information


**Supporting Information** Additional supporting information can be found online in the Supporting Information section.

## Data Availability

The data that support the findings of this study are available from the corresponding author upon reasonable request.
