# Supplementary material for: Association Between Socioeconomic Status and Mortality Risk in CKM Stage 0–3 Patients: Analysis of Inflammatory Mediation
Source: Cardiol Res Pract. 2026 Apr 12;2026:8849559. doi: 10.1155/crp/8849559 (PMC13071174; doi:10.1155/crp/8849559)
Supplement: Supplementary file 1 — Supporting Information Additional supporting information can be found online in the Supporting Information section. [file CRP-2026-8849559-s001.doc]

**Association Between Socioeconomic Status and Mortality Risk in CKM Stages 0-3 Patients: Analysis of Inflammatory Mediation**

Wenlong Ding^1,+^,Fachao Shi^3,+^,Lei Fang^4,+^,Qin Cui^4^,Zheng Wang^5,*^,Caoyang Fang^2,*^

^1^Department of Cardiology, Xuancheng Hospital Affiliated to Wannan Medical College (Xuancheng People 's Hospital), Xuancheng Anhui 242000, China;

^2^Department of Emergency,The First Affiliated Hospital of USTC,Division of Life Sciences and Medicine, University of Science and Technology of China, Hefei, Anhui,230000, China;

^3^Department of Cardiology,Maanshan People's Hospital,Maanshan Hospital Affiliated to Wannan Medical College,Maanshan, Anhui 243000, China;

^4^Department of Geriatrics Center,Tongling People's Hospital, Tongling, Anhui,244000,China;

^5^Department of Cardiology, The Second People's Hospital of Hefei, Hefei Hospital Affiliated to Anhui Medical University, Hefei, Anhui 230000, China;

***Corresponding Author:**

Zheng Wang,Department of Cardiology, The Second People's Hospital of Hefei, Hefei Hospital Affiliated to Anhui Medical University, Hefei, Anhui 230000, China;E-mail:wang20241007@163.com

Caoyang Fang,Department of Emergency,The First Affiliated Hospital of USTC,Division of Life Sciences and Medicine, University of Science and Technology of China, Hefei, Anhui,230000, China;E-mail:fcyahslyy@ustc.edu.cn

^+^:They contributed equally to the article.

**Table S1. Definition of CKM**

| CKM stages | Definition |
| --- | --- |
| CKM stage 0 | Participants with a normal Body Mass Index (BMI) (<23 kg/m² for those of Asian ethnicity and <25 kg/m² for individuals from other racial and ethnic backgrounds), and a normal waist circumference (<80 cm for Asian women and <90 cm for Asian men, or <88 cm for women and <102 cm for men in all other racial and ethnic groups) who did not fulfill the criteria for the other stages. |
| CKM stage 1 | Elevated Body Mass Index (BMI) (≥23 kg/m² for individuals of Asian descent and >25 kg/m² for all other racial and ethnic groups), increased waist circumference (≥80 cm for Asian women and ≥90 cm for Asian men, or ≥88 cm for women and ≥102 cm for men in other racial and ethnic categories), or prediabetes. Prediabetes is defined as a glycated hemoglobin (HbA1c) level of 5.7% to <6.5% or a fasting blood glucose level between 100 mg/dL and <126 mg/dL. |
| CKM stage 2 | Metabolic risk factors or moderate-to-high-risk chronic kidney disease (CKD) as defined by the Kidney Disease: Improving Global Outcomes (KDIGO) criteria, in accordance with AHA recommendations. The qualifying metabolic risk factors encompassed: elevated fasting serum triglycerides (≥135 mg/dL) ; hypertension; diabetes; metabolic syndrome, characterized by the presence of at least three of the following:  increased waist circumference; reduced high-density lipoprotein (HDL) cholesterol levels (<40 mg/dL for men, <50 mg/dL for women); fasting serum triglycerides ≥150 mg/dL; elevated blood pressure (systolic ≥130 mmHg, diastolic ≥80 mmHg, and/or use of antihypertensive medication); prediabetes. |
| CKM stage 3 | Presence of very-high-risk KDIGO CKD stages or a high-estimated 10-year cardiovascular disease (CVD) risk. The 10-year CVD risk was assessed using the AHA PREVENT equations for predicting cardiovascular events. A 10-year CVD risk of 20% or greater was classified as high risk. |
| CKM stage 4 | Reported history of established cardiovascular conditions, including coronary heart disease, angina, myocardial infarction, heart failure, and cerebrovascular accident. |

**Table S2.Sensitivity analysis excluding patients who died within 2 years prior to follow-up**

| **Variables** | **Model 1** | | **Model 2** | | **Model 3** | | **Model 4** | |
| --- | --- | --- | --- | --- | --- | --- | --- | --- |
|  | **HR(95%CI)** | **P** | **HR(95%CI)** | **P** | **HR(95%CI)** | **P** | **HR(95%CI)** | **P** |
| ***All-cause mortality*** | | | | | | | | |
| **PIR** | 0.85(0.81,0.88) | <0.0001 | 0.80(0.77,0.84) | <0.0001 | 0.88(0.84,0.93) | <0.0001 | 0.88(0.84,0.93) | <0.0001 |
| **SES** | | | | | | | | |
| **Low** | Ref | Ref | Ref | Ref | Ref | Ref | Ref | Ref |
| **Middle** | 0.91(0.77,1.08) | 0.30 | 0.65(0.56,0.76) | <0.0001 | 0.76(0.66,0.88) | <0.001 | 0.76(0.66,0.88) | <0.001 |
| **High** | 0.52(0.44,0.61) | <0.0001 | 0.41(0.35,0.50) | <0.0001 | 0.59(0.48,0.71) | <0.0001 | 0.59(0.49,0.72) | <0.0001 |
| **P for trend** | <0.0001 | | <0.0001 | | 0.006 | | 0.01 | |
| ***Cardiovascular mortality*** | | | | | | | | |
| **PIR** | 0.80(0.74,0.86) | <0.0001 | 0.75(0.68,0.82) | <0.0001 | 0.83(0.74,0.93) | 0.002 | 0.84(0.74,0.94) | 0.002 |
| **SES** | | | | | | | | |
| **Low** | Ref | Ref | Ref | Ref | Ref | Ref | Ref | Ref |
| **Middle** | 0.84(0.60,1.18) | 0.32 | 0.59(0.42,0.82) | 0.002 | 0.71(0.50,1.00 | 0.05 | 0.68(0.48,0.96) | 0.03 |
| **High** | 0.39(0.28,0.54) | <0.0001 | 0.31(0.21,0.44) | <0.0001 | 0.45(0.30,0.69) | <0.001 | 0.45(0.29,0.69) | <0.001 |
| **P for trend** | <0.0001 | <0.001 | 0.053 | 0.081 | <0.0001 | <0.001 | 0.053 | 0.081 |

**HR: hazard ratio, CI: confidence interval, Ref: reference**

**Model 1: No adjustments made;**

**Model 2: Adjusted for Age, Sex, Race;**

**Model 3:Adjusted for Age, Sex, Race,BMI,Marital,Education,Smoke,Alcohol**

**Model 4:Adjusted for Age, Sex, Race,BMI,Marital,Education,Smoke,Alcohol,eGFR,**

**HbA1c,TG,HDL,LDL,Drugs for hypertension, hyperlipidemia, diabetes**

**Table S3.Sensitivity analyses excluded participants with self-reported cancer diagnosis at baseline**

| **Variables** | **Model 1** | | **Model 2** | | **Model 3** | | **Model 4** | |
| --- | --- | --- | --- | --- | --- | --- | --- | --- |
|  | **HR(95%CI)** | **P** | **HR(95%CI)** | **P** | **HR(95%CI)** | **P** | **HR(95%CI)** | **P** |
| ***All-cause mortality*** | | | | | | | | |
| **PIR** | 0.84(0.80,0.88) | <0.0001 | 0.80(0.76,0.84) | <0.0001 | 0.89(0.84,0.94) | <0.0001 | 0.89(0.84,0.94) | <0.0001 |
| **SES** | | | | | | | | |
| **Low** | Ref | Ref | Ref | Ref | Ref | Ref | Ref | Ref |
| **Middle** | 0.84(0.71,0.99) | 0.04 | 0.66(0.56,0.77) | <0.0001 | 0.78(0.68,0.90) | <0.001 | 0.77(0.67,0.90) | <0.001 |
| **High** | 0.49(0.41,0.59) | <0.0001 | 0.41(0.34,0.50) | <0.0001 | 0.62(0.50,0.76) | <0.0001 | 0.61(0.50,0.76) | <0.0001 |
| **P for trend** | 0.635 | | 0.006 | | 0.05 | | 0.04 | |
| ***Cardiovascular mortality*** | | | | | | | | |
| **PIR** | 0.81(0.75,0.88) | <0.0001 | 0.77(0.69,0.85) | <0.0001 | 0.86(0.76,0.97) | 0.01 | 0.85(0.76,0.96) | 0.01 |
| **SES** | | | | | | | | |
| **Low** | Ref | Ref | Ref | Ref | Ref | Ref | Ref | Ref |
| **Middle** | 0.82(0.60,1.13) | 0.22 | 0.64(0.46,0.88) | 0.01 | 0.74(0.53,1.04) | 0.08 | 0.71(0.52,0.99) | 0.04 |
| **High** | 0.42(0.29,0.60) | <0.0001 | 0.35(0.24,0.51) | <0.0001 | 0.52(0.34,0.80) | 0.003 | 0.50(0.33,0.76) | 0.001 |
| **P for trend** | 0.833 | 0.205 | 0.437 | 0.318 | 0.833 | 0.205 | 0.437 | 0.318 |

**HR: hazard ratio, CI: confidence interval, Ref: reference**

**Model 1: No adjustments made;**

**Model 2: Adjusted for Age, Sex, Race;**

**Model 3:Adjusted for Age, Sex, Race,BMI,Marital,Education,Smoke,Alcohol**

**Model 4:Adjusted for Age, Sex, Race,BMI,Marital,Education,Smoke,Alcohol,eGFR,**

**HbA1c,TG,HDL,LDL,Drugs for hypertension, hyperlipidemia, diabetes**

**Table S4.Sensitivity Analysis Unweighted Data Analysis**

| **Variables** | **Model 1** | | **Model 2** | | **Model 3** | | **Model 4** | |
| --- | --- | --- | --- | --- | --- | --- | --- | --- |
|  | **HR(95%CI)** | **P** | **HR(95%CI)** | **P** | **HR(95%CI)** | **P** | **HR(95%CI)** | **P** |
| ***All-cause mortality*** | | | | | | | | |
| **PIR** | 0.87(0.84,0.89) | <0.0001 | 0.82(0.79,0.85) | <0.0001 | 0.88(0.85,0.92) | <0.0001 | 0.88(0.85,0.92) | <0.0001 |
| **SES** | | | | | | | | |
| **Low** | Ref | Ref | Ref | Ref | Ref | Ref | Ref | Ref |
| **Middle** | 0.90(0.81,1.00) | 0.06 | 0.68(0.61,0.76) | <0.0001 | 0.76(0.68,0.85) | <0.0001 | 0.76(0.68,0.85) | <0.0001 |
| **High** | 0.54(0.48,0.62) | <0.0001 | 0.45(0.40,0.52) | <0.0001 | 0.60(0.52,0.69) | <0.0001 | 0.60(0.52,0.70) | <0.0001 |
| **P for trend** | 0.323 | | <0.0001 | | <0.001 | | <0.001 | |
| ***Cardiovascular mortality*** | | | | | | | | |
| **PIR** | 0.83(0.78,0.88) | <0.0001 | 0.77(0.71,0.82) | <0.0001 | 0.84(0.78,0.91) | <0.0001 | 0.84(0.78,0.91) | <0.0001 |
| **SES** | | | | | | | | |
| **Low** | Ref | Ref | Ref | Ref | Ref | Ref | Ref | Ref |
| **Middle** | 0.94(0.75,1.17) | 0.58 | 0.68(0.54,0.85) | <0.001 | 0.80(0.63,1.01) | 0.06 | 0.78(0.62,0.99) | 0.04 |
| **High** | 0.45(0.34,0.59) | <0.0001 | 0.35(0.26,0.46) | <0.0001 | 0.49(0.36,0.67) | <0.0001 | 0.50(0.36,0.68) | <0.0001 |
| **P for trend** | 0.94 | 0.024 | 0.347 | 0.264 | 0.94 | 0.024 | 0.347 | 0.264 |

**HR: hazard ratio, CI: confidence interval, Ref: reference**

**Model 1: No adjustments made;**

**Model 2: Adjusted for Age, Sex, Race;**

**Model 3:Adjusted for Age, Sex, Race,BMI,Marital,Education,Smoke,Alcohol**

**Model 4:Adjusted for Age, Sex, Race,BMI,Marital,Education,Smoke,Alcohol,eGFR,**

**HbA1c,TG,HDL,LDL,Drugs for hypertension, hyperlipidemia, diabetes**
